# Tourscape, cultural identity, and loyalty in a themed cultural precinct: A study of Datang Everbright City in Xi’an, China

**DOI:** 10.1371/journal.pone.0358754

**Published:** 2026-09-24

**Authors:** Guibo Nie, Pengpeng Li, Mingtao Yan, Shaowei Ai, Jie He

**Affiliations:** 1 Key Research Institute of Yellow River Civilization and Sustainable Development & Collaborative Innovation Center on Yellow River Civilization, Henan University, Kaifeng, China; 2 China Tourism Academy (Data Center of the Ministry of Culture and Tourism), Beijing, China; 3 College of Geography and Environmental Science, Northwest Normal University, Lanzhou, China; 4 School of Management, Guangzhou University, Guangzhou, China; Sichuan Agricultural University, CHINA

## Abstract

The archaized block, a typical form of themed cultural precinct in China, creates a consumable and experiential “sense of history” through themed physical environments and symbolic performances. This type of urban space has emerged under the country’s cultural-tourism integration policy. In the field of cultural identity research on heritage sites with authentic historical remains, existing studies have confirmed the positive effects of factors such as authenticity of cultural heritage tourism, tourism experience, place attachment, and value perception on cultural identity. However, most of these studies presuppose the existence of objective historical relics. For themed cultural precincts like Datang Everbright City, which was developed without any authentic historical remains, the mechanism through which symbolic landscapes convey abstract historical meanings and stimulate tourists’ cultural identity remains underexplored in the academic literature. Drawing on constructive authenticity theory, and based on the S-O-R framework, this study constructs a theoretical model of how Tourscape perception influences cultural identity. A questionnaire survey was conducted among 289 tourists to Datang Everbright City and the data were analyzed using PLS-SEM. The study finds that cultural identity plays a significant mediating role between each dimension of Tourscape and destination loyalty, with full mediation for the physical and socio-symbolic dimensions and partial mediation for the social dimension. This study extends the application of Tourscape framework to the domain of “cultural identity”, validating its explanatory power in completely newly built themed cultural precincts, and examines the differentiated pathways through which each Tourscape dimension influences loyalty via cultural identity. These findings offer important theoretical insights into how themed cultural precincts carry historical meaning and stimulate cultural identity. They also provide practical guidance for cultural communication and experience design, as well as strategic references for destination managers seeking to enhance tourists’ cultural identity and revisit intentions.

## Introduction

Since the 1990s, with the deepening of China’s rapid urbanization and the intensification of competition between cities, the core dimensions of urban competitiveness have extended increasingly beyond traditional indicators such as economic output and industrial base. A growing body of evidence suggests that the development of urban cultural branding has become a key strategy in local government competition [1,2]. Against this backdrop, a wave of cultural precinct construction has swept across China. Local governments have increasingly engaged in selectively extracting and reinterpreting local historical resources as tourist attractions, collective memories, and cultural traditions, employing thematic marketing strategies to shape distinctive urban cultural brands and enhance tourism appeal. Oakes [3], in his study of rural cultural heritage display in China, further noted that in the pursuit of economic development, local governments often actively “thematize” historical resources to serve tourism development goals. This observation applies not only to rural contexts but also helps explain the logic behind the rapid development of cultural precincts.

Within the global landscape of urban competition and place marketing, cultural precincts in major Chinese cities have evolved into a distinct type of urban cultural tourism resource. From the integrated upgrading of the Nanjing Fuzimiao (Confucius Temple) area [4] to the tourism development of Chongqing Ciqikou Ancient Town [5], local governments have symbolically extracted and landscaped historical resources to shape unique urban cultural brands and enhance tourism attractiveness. This phenomenon has been widely discussed in academic research. Many scholars argue that within China’s macro-policy framework of urban renewal and heritage preservation, the renewal and construction of cultural precincts are regarded by the government as important spatial governance tools [6,7]. In this process, many precincts have been transformed into tourism spaces oriented toward cultural display and consumption, aiming to stimulate economic development through commercial operations [8]. However, compared with the large-scale construction boom, theoretical exploration of the cultural translation mechanisms of such precincts remains insufficient. The internal logic by which cultural precincts relate to tourists’ cultural identity urgently requires in-depth analysis.

In relevant academic discussions, the practice of cultural precincts in Chinese cities is generally classified into two interrelated yet distinct conceptual categories. “Historic cultural blocks” emphasize the authenticity and preservation value of spatial historical remains, with research focusing primarily on conservation strategies, authenticity maintenance, and community participation [9–12]. In contrast, “themed cultural precincts” refer to cultural tourism consumption spaces that are not based on authentic historical remains but are uniformly designed, constructed, and operated around a specific cultural theme. In the Chinese context, such spaces are often referred to as “archaized blocks”, characterized by the reproduction of historical styles in contemporary spaces, creating a consumable and experiential “sense of history” through the archaization of the physical environment [13]. The cultural meaning of such spaces is primarily constructed through cultural symbolism and experiential design, with research focusing on thematic narratives, tourist experiences, place identity, and cultural consumption [13,14]. To avoid ambiguity, the term “themed cultural precinct” is used uniformly hereafter.

Drawing on recent international tourism research, it is necessary to further clarify the conceptual distinction between themed cultural precincts and heritage destinations. A heritage destination is generally defined in tourism studies as a geographical space that possesses historical, cultural, or natural heritage resources and uses these resources as its core tourist attractions [15,16]. The two types of destinations differ fundamentally in their logic of heritage value attribution. The former represents a traditional heritage destination, whose cultural value is rooted in historical material remains, and tourists’ identification is built upon cognitive experiences of real history. In contrast, the heritage value of the latter derives from constructive authenticity, where the heritage referred to is not historical fact but rather a “meaningful sense of authenticity” constructed through symbolic extraction, thematic design, and narrative experiences, which tourists perceive as authentic [17]. Therefore, a themed cultural precinct can be regarded as a non‑traditional heritage destination.

Datang Everbright City Pedestrian Street in Xi’an is precisely such a non-traditional heritage destination. Themed around the Prosperous Tang Dynasty, it creates an open, immersive Tang cultural experience space through Tang-style architecture, large-scale thematic sculptures, panoramic light shows, and normalized performing arts IPs. Notably, all material elements of this precinct are contemporary constructions, containing no historical remains from the Tang Dynasty. In other words, the precinct does not protect an authentic historical relic but builds a “themed cultural landscape” from scratch in a new urban district. This completely reconstructed, non-heritage-based model of cultural production makes it a typical case for exploring how themed cultural precincts carry historical meaning and trigger tourists’ cultural identity.

To systematically address this issue, an analytical framework capable of integrating multiple elements is needed. The concept of Tourscape is precisely such a powerful tool. Proposed by Zhang and Xu [18], Tourscape aims to capture the overall environmental atmosphere experienced by tourists at a destination, integrating physical, social, socio-symbolic, and natural dimensions of stimuli, thus providing an integrated analytical framework for systematically deconstructing tourists’ environmental experiences. This framework has been applied to various tourism contexts. In ancient town tourism, Zhang and Xu [18] found that the physical and social dimensions of Tourscape are directly associated with liminal experiences, while the socio-symbolic and natural dimensions exert indirect effects through emotional arousal. In rural tourism, Chen et al. [19], based on the S-O-R framework, confirmed that rural tourism servicescapes influence tourists’ citizenship behavior through the mediating role of emotional and social value. In cultural heritage tourism, Torres-Moraga et al. [20] further found that multidimensional Tourscape is positively associated with tourists’ revisit intentions through the chain-mediated path of destination identification and trust.

These studies indicate that Tourscape, as an integrated analytical concept, demonstrates strong explanatory power across different tourism contexts. However, gaps remain in the existing literature. First, there has been insufficient typological extension of research objects. Existing research has primarily focused on two types of contexts: natural landscape destinations (e.g., national parks, seaside resorts) and historical heritage destinations (e.g., ancient cities, historic districts). However, themed cultural precincts like Datang Everbright City, which are entirely contemporary constructions, have received insufficient attention. The “sense of history” in such spaces is not naturally accumulated but is co-generated through the deliberate encoding of material carriers and tourists’ active decoding, with a cultural translation mechanism fundamentally different from that of authentic historical heritage sites [21,22]. Second, the mechanism through which Tourscape influences cultural identity remains unclear. Although existing research has confirmed that Tourscape, as a multidimensional environmental stimulus, can effectively influence tourists’ overall identification with a destination [20], how Tourscape specifically shapes the more culturally specific cultural identity—that is, the internal pathway through which individuals develop cognitive acceptance and emotional belonging to a particular cultural value system—has not been fully revealed. Accordingly, this study takes Xi’an Datang Everbright City as a case study, constructing a theoretical model incorporating both direct and mediating effects, to test the following hypothesized paths: (1) the direct effects of Tourscape dimensions on destination loyalty; (2) the effects of Tourscape dimensions on cultural identity; (3) the effect of cultural identity on destination loyalty; and (4) the mediating role of cultural identity between Tourscape dimensions and destination loyalty.

**Fig 1:**
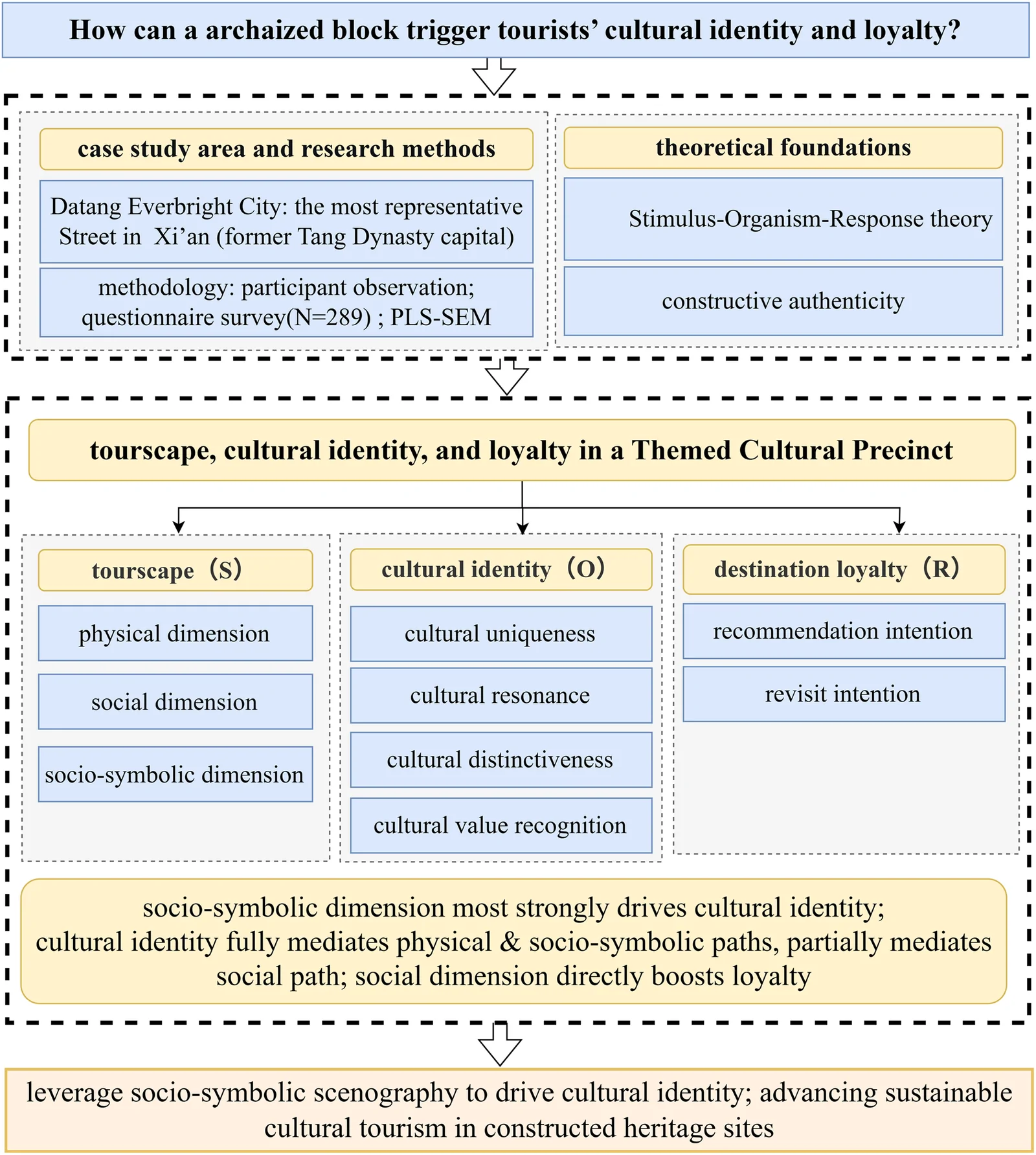
The research framework.

This study aims to make the following theoretical contributions. First, it extends the application of Tourscape framework to the domain of “cultural identity”, demonstrating its explanatory power in the specific context of themed cultural precincts without authentic historical remains. Second, drawing on constructive authenticity theory, it reveals the generative mechanism of cultural identity in themed cultural precincts, providing a new theoretical explanation for understanding “how modern landscapes carry historical meaning”. Third, it examines the mediating role of cultural identity between Tourscape and destination loyalty, revealing the differentiated pathways of different dimensions. Fig 1 presents the research framework in this study.

### Literature review and hypothesis development

This study adopts the Stimulus-Organism-Response (S-O-R) theory as its overall analytical framework. In this study, Tourscape constitutes the external environmental stimulus, cultural identity represents the tourists’ internal psychological state, and destination loyalty is the final behavioral response. The literature review and hypotheses are developed sequentially around this framework.

### Theoretical foundations

The S-O-R theory was proposed by Mehrabian and Russell [23], who argued that various stimuli in the external environment influence individuals’ internal psychological states, which in turn drive behavioral responses. This theory has been widely applied in fields such as consumer behavior, service marketing, and tourism research [23]. Existing studies provide important references for the framework of this paper. Wang and Li [24], taking three historic cultural blocks as cases, revealed the mechanism through which landscape visual quality influences destination loyalty via tourist emotions based on the S-O-R framework, finding that the visual quality of the built environment completely affects destination loyalty through the mediating role of tourist emotions. Liu et al. [25], taking the “most beautiful public cultural spaces” in Nanjing as a case, combined scene theory and the S-O-R framework and found that external environmental stimuli have both direct and indirect effects on tourists’ behavioral intentions, with cultural identity playing a mediating role, thereby supporting the “scene-identity-behavior” transmission path from the perspective of public cultural spaces.

Before testing the specific effects of each Tourscape dimension on cultural identity, it is necessary to theoretically clarify: why can an archaized block that is completely contemporarily built and contains no authentic Tang Dynasty remains still stimulate tourists’ identification with the Prosperous Tang culture? This study answers this question with the help of constructive authenticity theory. Wang [26] systematically distinguished three types of authenticity: objective authenticity, constructive authenticity, and existential authenticity. Among them, constructive authenticity is seen not as an inherent attribute of a tourism object but as generated through interaction between subject and object via symbolic systems and socio-cultural construction. In other words, something is perceived as authentic not because it objectively conforms to a historical fact, but because it is symbolically endowed with the signs and meanings of authenticity. In a completely newly built themed cultural precinct like Datang Everbright City, tourists experience a “Prosperous Tang atmosphere”. This atmosphere is constructed through the systematic extraction and creative recombination of Tang cultural symbols. Their authenticity perception is accomplished through the matching of cultural imagination with the symbolic landscape. This theoretical perspective provides important theoretical support for the subsequent hypotheses.

### Research hypotheses

The hypothesis development in this study is based on the S-O-R framework, incorporating both direct and mediating effect paths, testing sequentially: the direct effects of Tourscape dimensions on destination loyalty (H1a–H1c), the effects of Tourscape dimensions on cultural identity (H2a–H2c), the effect of cultural identity on destination loyalty (H3), and the mediating effect of cultural identity (H4).

1. Direct relationships between Tourscape dimensions and destination loyalty

The concept of Tourscape originates from servicescape in the service marketing field. Bitner [27] first proposed the concept of servicescape defining it as “the man-made, physical environment” and constructed an analytical framework comprising three dimensions—ambient conditions, spatial layout and functionality, and signs, symbols, and artifacts—to explain the effects of the physical environment on customers’ and employees’ cognition, emotion, and behavior. Subsequently, Baker et al. [28] incorporated social factors into the servicescape framework, emphasizing that the number, type, and behavior of people in the service environment also significantly affect customer experience. Rosenbaum and Massiah [29] further expanded this framework, proposing that servicescape should include four dimensions: physical dimension, social dimension, socio-symbolic dimension, and natural dimension. Among these, the socio-symbolic dimension focuses on symbols and signs that hold special meaning for particular groups, while the natural dimension focuses on the restorative effect of the environment on consumers’ psychology. Zhang and Xu [18] applied this framework to the field of tourism management, proposing the concept of Tourscape to capture the overall environmental atmosphere experienced by tourists at a destination. This expanded framework provides a more comprehensive analytical tool for understanding environmental stimuli in complex consumption spaces.

Destination loyalty refers to the psychological commitment and emotional preference that tourists exhibit toward a specific tourism destination, and the resulting stable behavioral tendency [30]. As a composite construct, destination loyalty typically comprises two core dimensions: revisit intention and recommendation intention [31]. Revisit intention reflects the continuity of the relationship between tourists and the destination, while recommendation intention reflects tourists’ tendency to translate their positive experiences into social dissemination behavior.

According to the S-O-R theoretical framework, external environmental stimuli are primarily associated with behavioral responses indirectly by influencing individuals’ internal psychological states. However, in some contexts, the environment itself may also trigger approach or avoidance behaviors. Harris and Ezeh [32] confirmed a direct statistical association between servicescape and loyalty intention, beyond mediation through satisfaction or emotional states. Therefore, there may be direct relationships between Tourscape dimensions and destination loyalty. Thus, this study proposes the following hypotheses:

H1a: The physical dimension of Tourscape positively affects destination loyalty.
H1b: The social dimension of Tourscape positively affects destination loyalty.
H1c: The socio-symbolic dimension of Tourscape positively affects destination loyalty.

2. Relationships between Tourscape and cultural identity

The concept of cultural identity can be traced back to social identity theory [33], which posits that individuals construct their self-concept through group membership, with ethnicity and culture serving as natural and significant dimensions of such categorization. Building upon this foundation, Phinney [34] defined cultural identity as a multidimensional construct encompassing self-identification, a sense of belonging, attitudes toward one’s ethnic group, and cultural participation. In the Chinese context, cultural identity is defined as the process by which individuals accept, recognize, and emotionally belong to the values, behavioral norms, and lifestyles of a particular cultural group. In the context of cultural heritage tourism, cultural identity is regarded as a core psychological outcome of tourists’ in-depth experience, marking the shift from external viewing and consumption to internal understanding and acceptance [35,36]. Existing studies have confirmed that Tourscape perception is an important antecedent of cultural identity construction. Zou et al. [37], using Quanzhou as a case and surveying 336 tourists, found that tourists’ heritage spatial perception significantly promotes their place identity, verifying that spatial perception is an important predictor of local cultural identity construction. Dai et al. [38], in a study of the Yellow River National Cultural Park, found that the generation of tourists’ cultural identity depends on the ecological traits of the landscape object and the constructed cultural representations. Zhang et al. [39], in a study of red tourism, found that material and spiritual cultural atmospheres can influence adolescents’ cultural identity. It should be noted that existing academic research often measures place identity or destination identity. However, cultural identity is conceptually distinct from these constructs. In environmental psychology, place identity has been defined as that component of an individual’s self-concept defined by the symbolic meaning of a particular physical environment or geographical space [40,41], with its core lying in individuals’ emotional connection to and sense of belonging with a specific place [42]. Tourism research has extended this logic by operationalizing destination identity as tourists’ perceptual identification with and emotional attachment to a specific destination space [37,43]. In contrast, cultural identity concerns individuals’ acceptance, recognition, and emotional affiliation with an abstract system of cultural values [35]. In the context of this study, the distinction is as follows: the former asks “how much I identify with this precinct itself”, while the latter asks “how much I identify with the Tang culture that this precinct represents”.

For themed cultural precincts, the formation mechanism of cultural identity has distinctive characteristics. The precinct is not a collection of authentic historical remains but a carefully constructed “accessible and experiential symbolic space of the Prosperous Tang” through the systematic extraction, creative recombination, and contemporary technological expression of Tang cultural symbols. Therefore, tourists’ cultural identity does not originate from a textual examination of historical authenticity but rather from the resonance and internalization of the cultural atmosphere, aesthetic style, and value narratives created in the immediate space. This mechanism can be explained through “constructive authenticity.” In themed cultural precincts such as Datang Everbright City, this constructive authenticity is particularly typical. Specifically, the physical dimension of Tourscape—such as Tang-style architecture, large sculptural groups, and light shows—serves as “signifiers,” directly and concretely evoking tourists’ collective imagination of the prosperous Tang era. The social dimension of Tourscape transforms tourists from passive landscape viewers into active participants. The socio-symbolic dimension—represented by themed businesses such as “Tang Food Square” and “Tang Ritual Square”—encodes abstract Tang cultural values into a system of observable, interactive, and consumable symbols, guiding tourists toward deep cultural resonance through daily visiting, consumption, and sharing practices. Based on the above theoretical analysis and literature review, this study proposes the following hypotheses:

H2a: Tourists’ perception of the physical dimension of Tourscape positively affects their cultural identity regarding the Tang culture presented at Datang Everbright City.
H2b: Tourists’ perception of the social dimension of Tourscape positively affects their cultural identity regarding the Tang culture presented at Datang Everbright City.
H2c: Tourists’ perception of the socio-symbolic dimension of Tourscape positively affects their cultural identity regarding the Tang culture presented at Datang Everbright City.

3. Relationship between cultural identity and destination loyalty

Based on the S-O-R theoretical framework, external environmental stimuli are primarily associated with behavioral responses by influencing individuals’ internal psychological states, and each dimension of Tourscape triggers loyalty behavior outcomes by activating tourists’ identification with Tang culture. Cultural identity, as an internal psychological state formed by tourists under external environmental stimuli, constitutes a key link between Tourscape stimuli and tourists’ behavioral responses. In highly symbolic consumption spaces such as themed cultural precincts, tourists’ identification with Tang culture is essentially a form of symbolic identity—that is, confirming and strengthening one’s cultural identity through the consumption and display of symbols related to the Tang Dynasty. In the Chinese cultural context, recent empirical studies provide multiple perspectives of evidence for this relationship. Fu and Luo [35], in a study of the Liangzhu Ancient City site, also found a significant positive relationship between cultural identity and revisit intention. Qin and Zhang [44], in a study of cultural heritage tourism in Ningxia, similarly found that cultural identity is a key mediating variable connecting rich cultural tourism experiences and behavioral intentions, with a stable effect. Dong and Yahaya [45], in a heritage tourism study centered on Shandong Province, found that cultural identity is positively associated with tourists’ behavioral intentions. Liu et al. [46], using the Mount Tai heritage site as a sample, further showed that among multiple antecedents including cultural identity, experience quality, and perceived value, cultural identity is the strongest predictor of tourists’ revisit intention. In the field of cultural leisure consumption, research has also confirmed that cultural identity is an important predictor of building consumer loyalty [47]. The above studies, taking different cultural contexts and heritage types, consistently show that cultural identity is a key psychological antecedent of tourist loyalty. Based on the above theoretical analysis, this study proposes:

H3: Tourists’ cultural identity positively affects their destination loyalty.

4. The mediating role of cultural identity

Under the S-O-R framework, external environmental stimuli do not directly determine behavioral responses but rather exert influence indirectly through affecting individuals’ internal psychological states. Tourscape, as an external environmental stimulus, has a significant positive effect on cultural identity. Cultural identity, as an internal psychological state, has a significant positive effect on destination loyalty.

Furthermore, cultural identity may also mediate the relationship between Tourscape and destination loyalty. Qin and Zhang [44], in a study of cultural heritage sites in Ningxia, found that cultural identity plays a significant mediating role between rich cultural tourism experiences and satisfaction. Du and Wang [48], using the Zhongshan Road and Sifang Road historic districts in Qingdao as cases, employed qualitative analysis and structural equation modeling to reveal that place identity partially mediates the relationship between perceived historical and entertainment value and behavioral intention. Although the research contexts vary, these studies collectively indicate that in cultural tourism experiences, tourists’ cultural identity toward the destination is a key psychological mechanism connecting environmental stimuli and destination loyalty. Based on the above deduction, this study proposes:

H4: Cultural identity mediates the relationship between Tourscape and destination loyalty.

### Theoretical model

**Fig 2:**
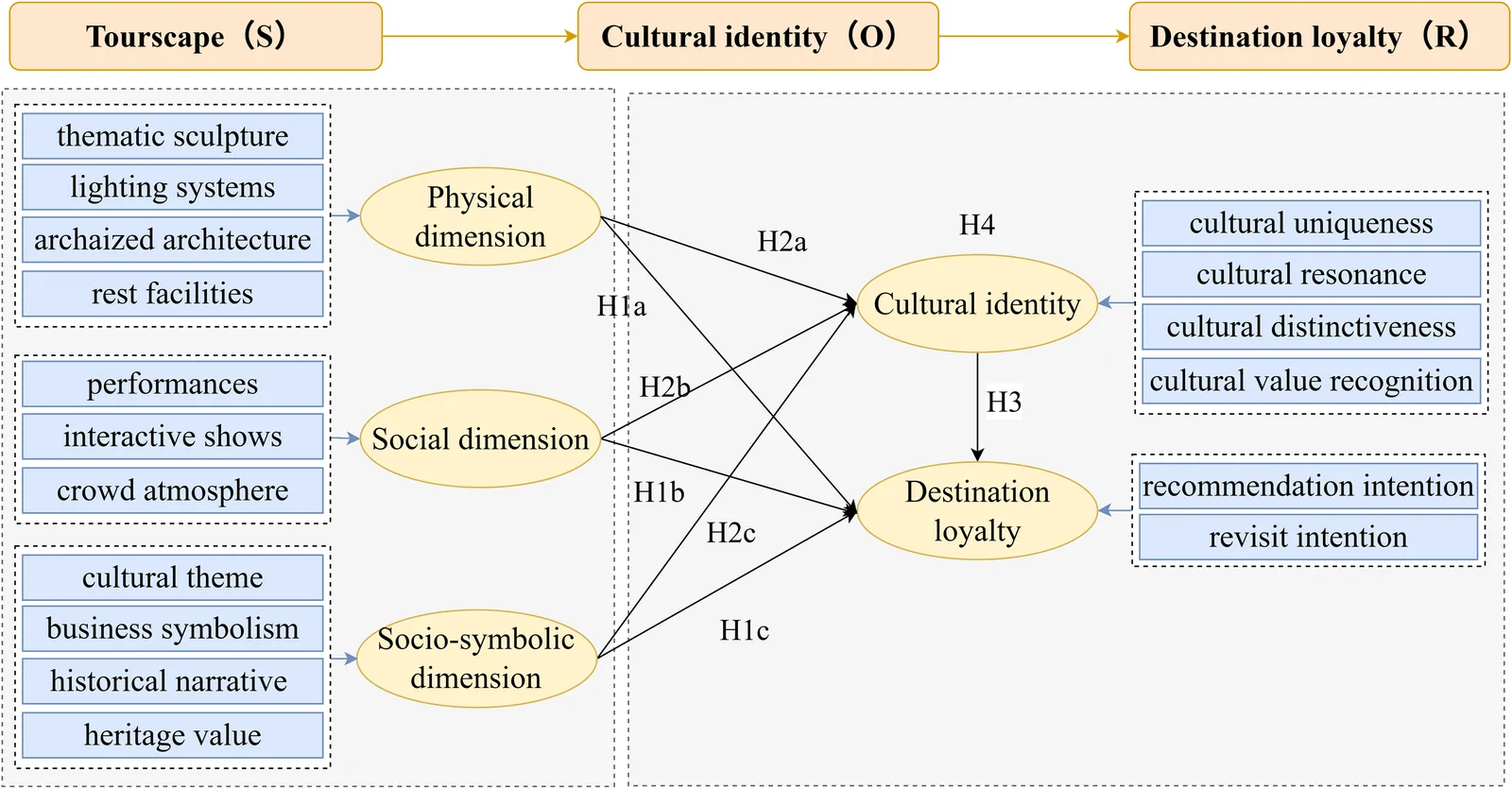
Theoretical model hypotheses.

Combining the above hypotheses, the theoretical model constructed in this study is shown in Fig 2.

## Methods

### Case study area

**Fig 3:**
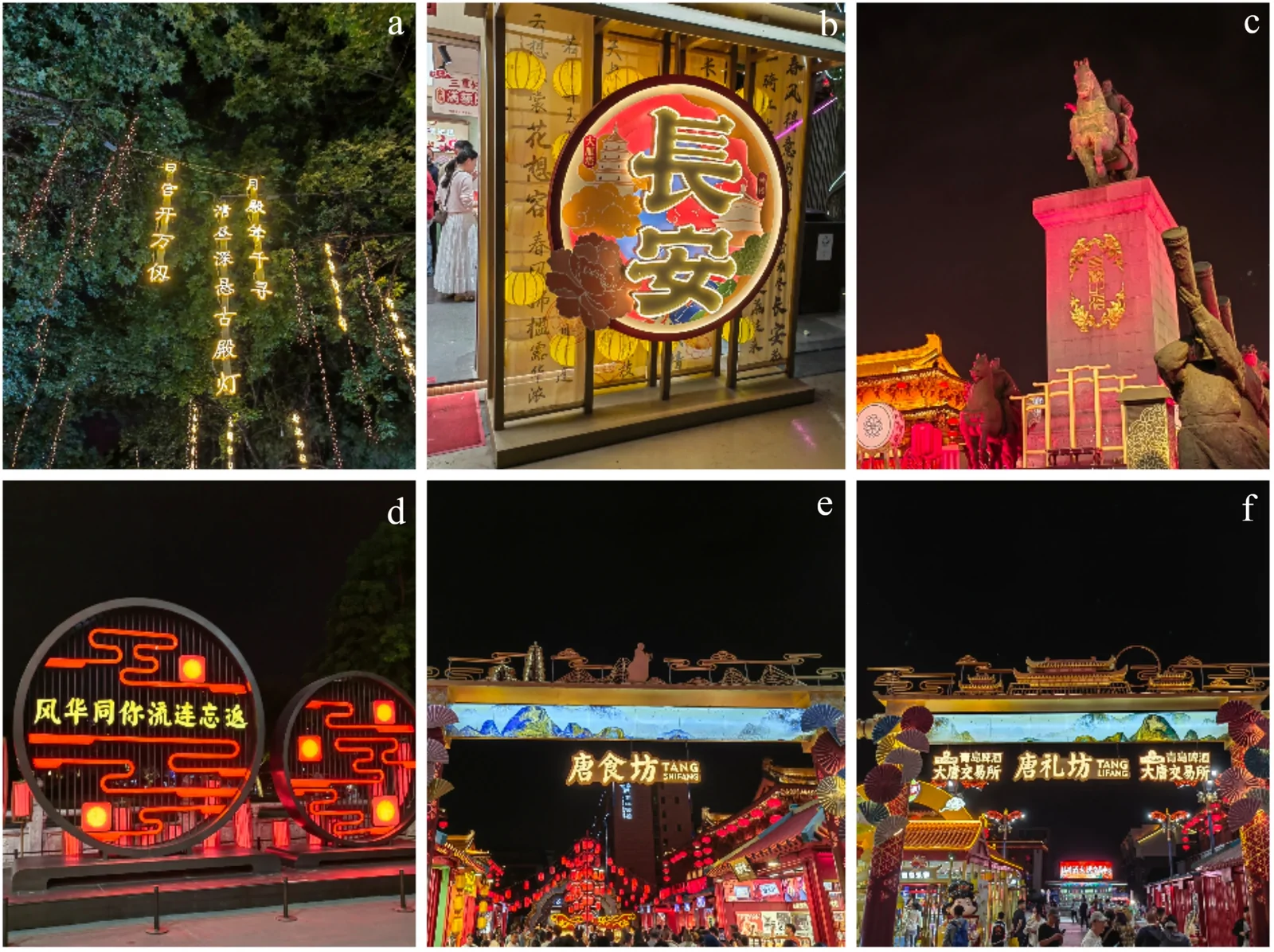
Featured scenes of Datang Everbright City Pedestrian Street. (a) The Poem-decorated light belts; (b) Tang-style shop windows; (c) The “Prosperous Reign of Zhenguan” thematic sculpture group; (d) Photo check-in spots within the precinct; (e) & (f) Themed business clusters of “Tang Food Square” and “Tang Ritual Square”.

Datang Everbright City Pedestrian Street is located in Yanta District, Xi’an, Shaanxi Province, China (Fig 3). It spans from the South Square of the Giant Wild Goose Pagoda in the north to the Tang City Wall Ruins Park in the south, spanning approximately 2,100 meters in length and covering about 1.2 square kilometers. All buildings, sculptures, lighting installations, and landscape facilities in this precinct were constructed in the 21st century and contain no historical remains from the Tang Dynasty. The precinct is themed around Prosperous Tang Culture, creating an immersive Tang cultural experience space through symbolic means such as Tang-style architecture, large-scale thematic sculptures, panoramic lighting systems, and normalized street performances.

Datang Everbright City was selected as the case study for three main reasons. First, it represents a typical type of themed cultural precinct, making it an ideal context for testing the explanatory power of Tourscape framework in constructed cultural spaces. Second, it is a national-level tourism and leisure precinct and a national demonstration pedestrian street, serving as a benchmark in China’s cultural tourism industry with certain typicality and representativeness. Third, its enormous visitor numbers provide a sufficient sample base for the questionnaire survey.

### Data collection process

Data collection for the case site followed three steps. First, through government websites such as the Xi’an Municipal Bureau of Culture and Tourism and the Qujiang New District Management Committee, as well as official WeChat public accounts such as Xi’an Culture and Tourism, Qujiang Cultural Industry Group, and Datang Everbright City official, relevant planning documents, policy bulletins, news reports, and other textual materials were systematically collected to obtain a comprehensive understanding of the study site. On this basis, scales were designed following the Tourscape framework’s dimensions: physical, social, socio-symbolic, and natural. Second, the research team conducted a pre-survey at Datang Everbright City in December 2025. A total of 50 questionnaires were distributed to tourists after their visit, and 47 valid questionnaires were returned. Based on the pre-survey results, the wording of some items was slightly adjusted, and the natural dimension was removed to ensure that respondents accurately understood the meaning of the items. Pre-test data were excluded from the final analysis. Third, a formal survey was conducted at Datang Everbright City Pedestrian Street in February 2026. Datang Everbright City Pedestrian Street is a fully public pedestrian street, open to all visitors without an admission fee, so no formal permit was required for conducting the survey. Nevertheless, prior to the formal survey, the research team submitted a formal request to the Xi’an Qujiang New District Management Committee (the administrative authority of the precinct), describing the study’s objectives and data collection procedures. The formal survey employed an on-site random interception method. All questionnaires were distributed and completed within the Datang Everbright City precinct. Respondents completed electronic questionnaires on-site using mobile devices provided by the researchers (with the Tencent Wenjuan platform serving as the electronic data-entry interface), while paper questionnaires were offered to those who preferred not to use electronic devices. Each visitor was limited to one response, and no remotely completed questionnaires were included in the final dataset. Data were collected during two periods: December 12–13, 2025 (pre-survey) and February 10–15, 2026 (formal survey).In addition, to assist in questionnaire design and deepen understanding of the case site, the research team conducted informal participatory observations during field visits and had brief conversations with some tourists and merchants. These exploratory activities provided contextual information for the finalization of the questionnaire, and their results were not systematically analyzed as formal data.

It should be noted that the two time points for field research (December 2025 and February 2026) were chosen because the precinct’s performances are adjusted according to peak and off-peak seasons. During the peak season, the visitor flow is high and performance activities are dense, allowing for a more comprehensive observation of tourists’ perceptions of different dimensions of Tourscape.

This study was not medical research and did not involve experimentation on human subjects. In accordance with the principles of the Declaration of Helsinki and Article 32 of China’s Ethical Review Measures for Life Sciences and Medical Research Involving Humans (2023), this non-interventional behavioral research qualified for exemption from formal ethical review. The researchers provided all participants with a written informed consent information sheet, and the researchers orally explained the research purpose, data usage, anonymization procedures, and the right to withdraw. Participation was voluntary, and completion and submission of the questionnaire constituted informed consent; participants were not required to sign an informed consent form. For the semi-structured interviews, separate oral consent was obtained from each participant before the interview commenced, following the same informed-consent standards. All data were anonymized, and no personally identifiable information was collected. Data have been used solely for research purposes.

### Data analysis method

This study applied Partial Least Squares Structural Equation Modeling (PLS-SEM) using SmartPLS 4.0. PLS-SEM was chosen for two reasons: first, PLS-SEM has less strict assumptions regarding data normality, making it suitable for actual data in the social sciences; second, PLS-SEM focuses on predicting and explaining variance, aligning with the objectives of this study.

The analysis procedure followed the recommendations of Hair et al. [49]. First, the measurement model was evaluated, testing the internal consistency reliability, convergent validity, and discriminant validity of each latent variable. Second, the structural model was evaluated, testing path coefficients, coefficients of determination (R^2^), effect sizes (f^2^), and predictive relevance (Q^2^).

### Measures

All constructs were measured using a five-point Likert scale, where 1 represented “strongly disagree” and 5 represented “strongly agree.” The scales were translated using a back-translation procedure to ensure equivalence between the Chinese and English versions: first, the original English scales were translated into Chinese by the researcher, and then back-translated into English by another researcher proficient in English; discrepancies were resolved through discussion and revision. We also implemented the following procedural measures during the questionnaire design stage to reduce the potential impact of common method bias: (a) the questionnaire was administered anonymously; (b) the order of items was randomized while maintaining logical coherence; (c) respondents were reminded that “there are no right or wrong answers, please respond according to your true feelings”.

The Tourscape measurement items were adapted from scales developed by Zhang and Xu [18] and Rosenbaum and Massiah [29]. In the pre-survey, we measured items across all four dimensions. The standardized factor loadings for all items in the natural dimension were below the recommended threshold of 0.50. This suggests that, in our research context, the natural dimension may not be a core source of tourists’ environmental perception. Moreover, given the spatial attributes of Datang Everbright City as a modern urban themed cultural precinct, its environment is predominantly composed of artificial architecture, hard paving, and landscape structures, with scarce natural landscape elements. Field observations also indicate the marginal role of natural elements in this precinct (see Fig 3).

Therefore, to concentrate on the core influence mechanism and enhance the model’s explanatory power, we made a context-specific selection of Tourscape dimensions in constructing our analytical framework. The final Tourscape measurement items comprised the following three dimensions. The physical dimension included four items measuring tourists’ perception of material environments such as thematic sculptures, lighting systems, archaized architecture, and rest facilities. The social dimension included three items measuring perception of social environments such as performances, interactive shows, and crowd atmosphere. The socio-symbolic dimension included four items measuring perception of cultural theme expression, symbolic meanings of businesses, historical narrative implications, and cultural heritage value. Notably, the measurement of the social dimension focused on interaction with performers and NPCs, based on the contextual characteristics of Datang Everbright City as a completely newly built archaized block with no original resident population and featuring normalized performances as its core attraction.

The cultural identity measurement items were adapted from scales developed by Fu and Luo [35] and Yang et al. [36], measuring cultural uniqueness, cultural resonance, cultural distinctiveness, and cultural value recognition, with a total of four items. Destination loyalty was comprehensively measured using recommendation intention and revisit intention, drawing on classic construct scales such as Yoon and Uysal [31] and Chi and Qu [50]. This two-item measurement paradigm has a long-standing and broad academic foundation in tourism research and has been consistently adopted by numerous empirical studies across diverse contexts, including religious festival tourism [51], ecotourism [52], island tourism [53], cultural tourism [54], and sustainable tourism [55]. The measurement items are shown in Table 1

**Table 1:** The measurement items.

Construct	Dimension	Code	Item
**Tourscape**	**Physical dimension**	PHY1	The large thematic sculpture groups such as “The Prosperous Reign of Zhenguan” are grand in scale and exquisitely carved.
		PHY2	The nighttime light and shadow effects are rich in color and clearly layered, creating a unique nighttime visual environment.
		PHY3	The Tang-style architecture of the block blends well with modern design elements, creating a visually coordinated and unified effect.
		PHY4	The rest areas within the block are reasonably arranged, making it convenient to stop and rest at any time.
	**Social dimension**	SOC1	The performances of the Tang Dynasty Lady are wonderful and interesting, attracting many tourists to stop and watch.
		SOC2	Various interactive activities give me the opportunity to integrate into the performance scene rather than just watching from the sidelines.
		SOC3	The lively atmosphere of photo-taking and social media sharing within the block encourages me to participate more actively in the on-site interactions.
	**Socio-symbolic dimension**	SSY1	The naming and design of themed businesses such as Tang Food Square and Tang Ritual Square reflect the symbolic characteristics of the Prosperous Tang culture.
		SSY2	The unique flavors of time-honored brands such as Tong Sheng Xiang and De Fa Chang within the block allow me to feel the heritage of Tang Dynasty food culture.
		SSY3	The cultural and creative products are exquisitely designed, cleverly integrating Tang cultural elements on the basis of a new Chinese aesthetic.
		SSY4	The cultural theme of the Prosperous Tang atmosphere is expressed uniformly through architecture, performances, and business formats.
**Cultural identity**		CI1	For me, the Tang culture experience provided here is something I cannot feel elsewhere.
		CI2	The distinctive performances, architecture, and light and shadow in the block deepen my understanding of and resonance with the Tang culture.
		CI3	Compared with other blocks, I think the cultural style presented here has a distinctive recognizability.
		CI4	Emotionally, I identify with and appreciate the Tang culture information conveyed here.
**Destination loyalty**	**Recommendation intention**	DL1	I am willing to recommend this place to family and friends as a destination to learn about Tang culture.
	**Revisit intention**	DL2	If I have the opportunity in the future, I am willing to visit here again.

In addition, the questionnaire collected demographic information such as gender, age, occupation, education level, place of residence, number of visits, as well as travel motivation as auxiliary variables.

## Research results

### Sample characteristics

In the formal survey, a total of 350 questionnaires were distributed. After excluding questionnaires completed in less than 90 seconds, straight-line responses and logical inconsistencies in reverse-coded items, 289 valid questionnaires were obtained, with an effective rate of 82.57%. The demographic characteristics of the valid sample are shown in Table 2.

**Table 2:** Sample demographic characteristics.

Indicator	Category	Count	Percentage
Gender	Male	125	43.25%
	Female	164	56.75%
Occupation	Student	97	33.56%
	Enterprise employee	82	28.37%
	Government/institution employee	68	23.53%
	Self-employed	2	0.69%
	Freelancer	34	11.76%
	Retired	2	0.69%
	Other	4	1.38%
Education	Junior high school or below	4	1.38%
	High school/vocational school	6	2.08%
	Associate/Bachelor’s degree	150	51.90%
	Postgraduate or above	129	44.64%
Age	18–25 years	97	33.57%
	26–35 years	154	53.29%
	36–50 years	34	11.76%
	51–65 years	4	1.38%
	66 years and above	0	0.00%
Residence	Xi’an city	34	11.76%
	Shaanxi Province (outside Xi’an)	8	2.77%
	Other provinces	247	85.47%
Number of visits	First time	189	65.40%
	2–3 times	74	25.61%
	4 times or more	26	9.00%

The proportion of female respondents was slightly higher than that of males. The age distribution was dominated by young and middle-aged adults, with the 26–35 age group accounting for the highest proportion, followed by the 18–25 age group. Together, these two groups comprised over 86.86% of the sample. In terms of occupation, students accounted for 33.56%, enterprise employees 28.37%, and government/institution employees 23.53%. The overall educational level was relatively high, with those holding an associate/bachelor’s degree or above accounting for 96.54% of the sample. The distribution of residence showed that 85.47% of tourists came from outside Shaanxi Province, indicating strong appeal to non-local tourists. Regarding the number of visits, first-time visitors accounted for 65.40%, those visiting 2–3 times accounted for 25.61%, and those visiting 4 times or more accounted for 9.00%.

### Reliability and validity

Before testing the structural model, the reliability and validity of the measurement model were evaluated. All analyses were conducted using SmartPLS 4.0 with 5,000 bootstrap resamples.

1. **Internal consistency reliability and convergent validity**

As shown in Table 3, the composite reliability (CR) of each latent variable ranged from 0.916 to 0.958, and Cronbach’s α ranged from 0.863 to 0.924, both exceeding the recommended threshold of 0.70, indicating good internal consistency reliability. The standardized factor loadings of all measurement items ranged from 0.817 to 0.959, all greater than 0.70 and significant at the *p* < 0.001 level. The average variance extracted (AVE) for each latent variable ranged from 0.733 to 0.919, all exceeding the standard of 0.50, indicating good convergent validity.

**Table 3:** Reliability and convergent validity results.

Latent variable	Item	Factor loading	Cronbach’s α	CR	AVE
**Physical dimension**	PHY1	0.832	0.878	0.916	0.733
	PHY2	0.880			
	PHY3	0.894			
	PHY4	0.856			
**Social dimension**	SOC1	0.817	0.863	0.916	0.785
	SOC2	0.885			
	SOC3	0.910			
**Socio-symbolic dimension**	SSY1	0.863	0.898	0.929	0.766
	SSY2	0.878			
	SSY3	0.895			
	SSY4	0.873			
**Cultural identity**	CI1	0.887	0.924	0.946	0.815
	CI2	0.925			
	CI3	0.894			
	CI4	0.904			
**Destination loyalty**	DL1	0.959	0.911	0.958	0.919
	DL2	0.957

2. **Discriminant validity**

Discriminant validity was tested using the HTMT criterion [56]. As shown in Table 4, all HTMT values were below the recommended threshold of 0.90, indicating acceptable discriminant validity. Among them, the HTMT value between the social and socio-symbolic dimensions was 0.871, slightly above the conservative threshold of 0.85 but still below the more lenient threshold of 0.90. This high correlation reflects the integrated nature of themed cultural precincts, where social interactions and symbolic elements are deliberately intertwined. The Fornell-Larcker criterion was also satisfied, further supporting the distinctiveness of the constructs.

**Table 4:** Discriminant validity (HTMT ratios).

Variable	Physical dimension	Social dimension	Socio-symbolic dimension	Cultural identity	Loyalty
Physical dimension					
Social dimension	0.764				
Socio-symbolic dimension	0.759	0.871			
Cultural identity	0.792	0.789	0.806		
Loyalty	0.825	0.769	0.741	0.801	

*Note: All values below 0.90 indicate acceptable discriminant validity.*

Since all variables were collected through the same questionnaire, common method bias (CMB) may be a concern. To assess this, we first conducted Harman’s single-factor test. The results showed that the first unrotated factor explained 44.865% of the total variance, which is below the 50% threshold [49,57], indicating that no single dominant factor accounted for the majority of the variance. Second, we further tested using the Unmeasured Latent Method Construct (ULMC) approach [58]. The results showed that the Satorra-Bentler scaled chi-square difference test was not significant, and the changes in model fit indices were ΔCFI = 0.002, ΔRMSEA<0.001, and ΔSRMR = 0.002, all below the recommended threshold of 0.01. The above results indicate that common method bias is not a serious issue in this study. However, given the inherent limitations of Harman’s test as a post hoc diagnostic method and the data collection approach of this study, CMB cannot be completely ruled out.

### Path analysis

After confirming the measurement model, the structural model was tested. The variance inflation factor (VIF) for all predictor variables was below the critical value of 5.0, indicating no serious multicollinearity issues.

1. Hypothesis testing

PLS-SEM was used to test the hypothesized paths, with bootstrapping to estimate the significance of path coefficients and bias-corrected confidence intervals.Based on the path coefficients and hypothesis testing results in Table 5, five of the seven proposed hypotheses received empirical support, yielding a support rate of 71.4%.

**Table 5:** Structural model path coefficients and hypothesis testing.

Hypothesis	Path relationship	Path coefficient (β)	t-value	p-value	95% CI	Result
H1a	Physical dimension → Destination loyalty	0.033	0.331	0.740	[-0.146, 0.241]	Not supported
H1b	Social dimension → Destination loyalty	0.264*	2.358	0.018	[0.042, 0.477]	Supported
H1c	Socio-symbolic dimension → Destination loyalty	0.061	0.586	0.558	[-0.154, 0.258]	Not supported
H2a	Physical dimension → Cultural identity	0.279**	3.029	0.002	[0.094, 0.456]	Supported
H2b	Social dimension → Cultural identity	0.225**	2.832	0.005	[0.074, 0.384]	Supported
H2c	Socio-symbolic dimension → Cultural identity	0.396***	4.271	<0.001	[0.218, 0.583]	Supported
H3	Cultural identity → Destination loyalty	0.540***	6.883	<0.001	[0.387, 0.699]	Supported

Note: Bootstrap resampling = 5,000 times; CI = bias-corrected 95% confidence interval. *p < 0.05, **p < 0.01, ***p < 0.001.

Specifically, among the direct effects, only the direct effect of the social dimension was significant, supporting H1b; the direct effects of the physical and socio-symbolic dimensions were not significant, so H1a and H1c were not supported. For the paths from Tourscape dimensions to cultural identity, the physical, social, and socio-symbolic dimensions all had significant positive effects on cultural identity, supporting H2a, H2b, and H2c. Among these, the socio-symbolic dimension had the largest path coefficient, indicating that culturally symbolic elements exert the strongest effect; the physical and social dimensions also played positive roles, but with relatively weaker effects. For the path from cultural identity to destination loyalty, cultural identity had a significant positive effect on destination loyalty, supporting H3, indicating that tourists’ psychological identification with Tang culture is the key psychological mechanism associated with revisit and recommendation intentions.

In summary, the results confirm that, except for the direct paths of the physical and socio-symbolic dimensions, all the other dimensions are significantly associated with destination loyalty by strengthening tourists’ cultural identity. Cultural identity fully mediates the relationships between the physical dimension and destination loyalty and between the socio-symbolic dimension and destination loyalty, and partially mediates the relationship between the social dimension and destination loyalty.

After testing the significance of path coefficients and hypotheses, the overall explanatory power and predictive ability of the structural model were systematically assessed. The *R*^2^ value for cultural identity was 0.732, indicating that the three environmental elements (physical, social, and socio-symbolic dimensions) together explained 73.2% of the variance in tourists’ cultural identity, well above the moderate threshold of 0.50, reaching a strong level and demonstrating excellent explanatory power for the formation of tourists’ cultural identity. The *R*^2^ value for destination loyalty was 0.722, indicating that cultural identity as the core psychological mediating mechanism, together with the direct and indirect effects of Tourscape dimensions, explained 72.2% of the variance in destination loyalty, confirming the strong predictive power of the model.

2. Mediating effect test

The bootstrap method was used to test the mediating effect of cultural identity. As shown in Table 6, all three specific indirect paths reached significance. Specifically, the indirect effect of the socio-symbolic dimension on destination loyalty through cultural identity was the strongest, indicating that symbolic elements such as cultural symbols, creative products, and themed businesses in the precinct re positively associated with tourists’ psychological identification with Tang culture, which in turn are associated with their revisit and recommendation intentions. The indirect effect of the physical dimension on destination loyalty through cultural identity was 0.151, indicating that perception of the landscape features, such as large thematic sculptures, light shows, and archaized architecture, is primarily transformed into behavioral intentions via the cognitive path of identity. The indirect effect of the social dimension through cultural identity was 0.122, which, although relatively weaker, was still significant, indicating that social elements such as street performances and interactive experiences are indirectly associated with tourists’ behavioral intentions by strengthening cultural identity. Combined with the direct effect results, it can be concluded that the direct effects of the physical and socio-symbolic dimensions on destination loyalty are not significant, whereas their indirect effects are significant, indicating that cultural identity plays a fully mediating role. The direct and indirect effects of the social dimension are both significant, indicating a partial mediating role. These results support Hypothesis H4, namely that cultural identity mediates the relationship between each Tourscape dimension and destination loyalty, with differences in the type of mediation across dimensions. This reflects that, in the immersive Tang cultural space of Datang Everbright City, symbolic and representational elements are the key channel through which tourists form cultural identity and subsequently generate behavioral intentions.

**Table 6:** Mediating effect test results.

Mediating path	Effect value	Standard error	t-value	p-value	95% CI	Conclusion
Physical dimension → Cultural identity → Destination loyalty	0.151	0.05	3.034	0.002	[0.054, 0.249]	Full mediation
Social dimension → Cultural identity → Destination loyalty	0.122	0.05	2.422	0.015	[0.037, 0.231]	Partial mediation
Socio-symbolic dimension → Cultural identity → Destination loyalty	0.214	0.064	3.365	0.001	[0.107, 0.354]	Full mediation

*Note: Mediation type was determined based on the significance of both direct and indirect paths. Full mediation is concluded when the indirect path is significant but the direct path is not; partial mediation is concluded when both paths are significant.*

**Fig 4:**
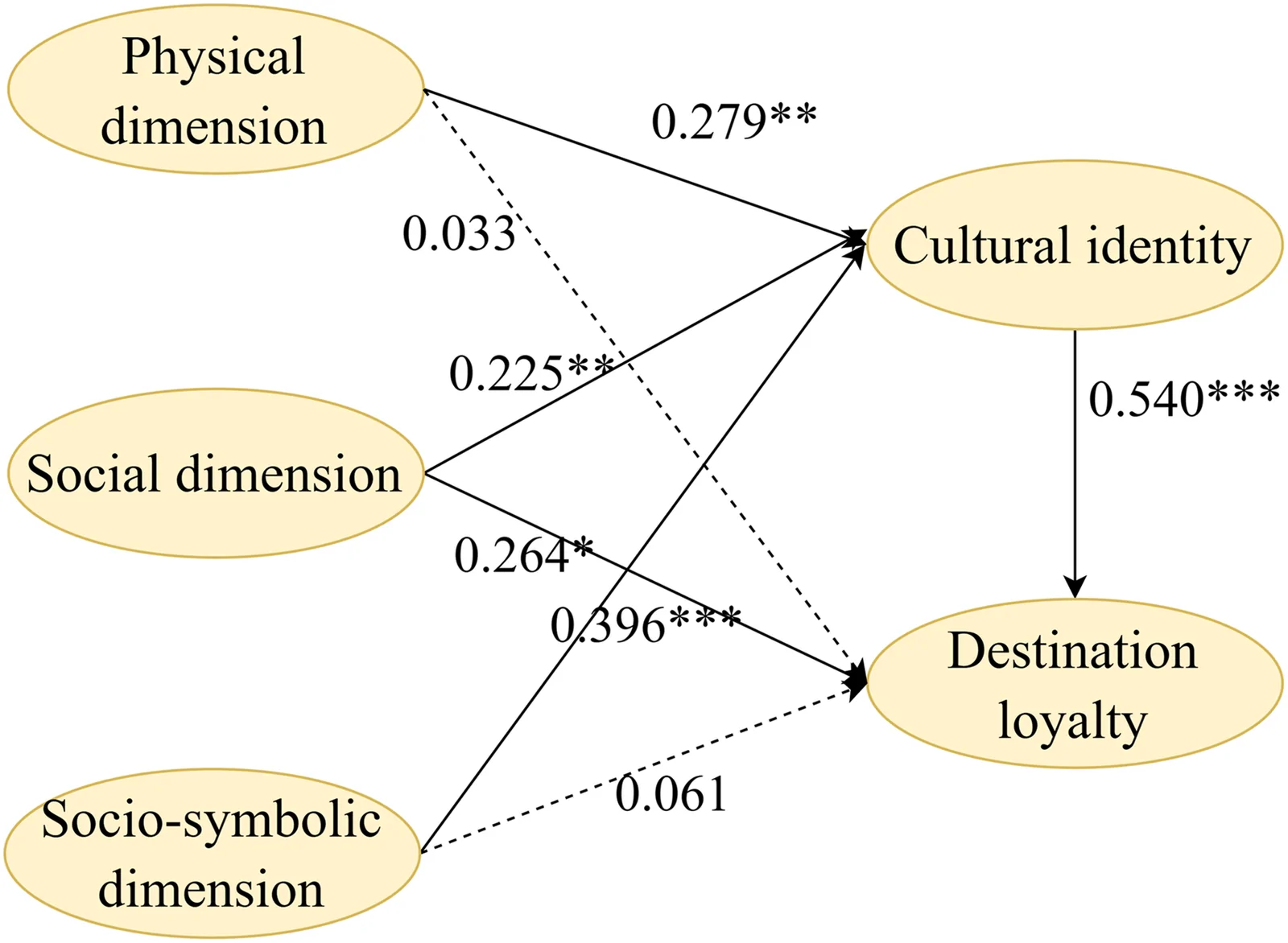
Path diagram of the structural equation model. Solid lines indicate significant paths (*p* < 0.05); dashed lines indicate non-significant paths (p≥0.05). ***p<0.001, **p<0.01, *p<0.05.

To more intuitively present the mechanism of influence of each Tourscape dimension on tourists’ behavioral intentions, a path diagram of the structural equation model is presented. As shown in Fig 4, the paths from Tourscape dimensions to destination loyalty show differentiated characteristics. The indirect effect of the socio-symbolic dimension on destination loyalty through cultural identity is the strongest, indicating that symbolic elements such as cultural symbols, creative products, and themed businesses are the most effective in stimulating tourists’ psychological identification with Tang culture, which in turn are associated with revisit and recommendation intentions, and are the core channel for the generation of cultural identity. The physical dimension also exerts a fully mediating role through cultural identity, with no significant direct effect. The social dimension exhibits partial mediation, not only indirectly influencing destination loyalty through cultural identity but also directly and positively associated with tourists’ behavioral intentions.

## Discussion

### Theoretical implications

This study contributes to the literature in three key ways.

First, this study extends the explanatory scope of the Tourscape framework to the domain of “cultural identity”, further revealing the role of Tourscape in the formation of cultural identity. Existing Tourscape research has primarily focused on its influence on psychological and behavioral outcomes such as place attachment [59], liminal experience [18], and destination loyalty [20], with most of these studies conducted in heritage tourism contexts. However, Datang Everbright City, as a newly built themed cultural precinct, lacks authentic historical remains. Its authenticity is primarily constructed through the atmosphere created by symbolically coded cultural elements, and what this atmosphere conveys is a constructed and communicated system of abstract cultural values. For themed cultural precincts centered on cultural experience, various Tourscape dimensions are not merely environmental backdrops that carry tourism experiences but are important media for transmitting cultural values; tourists’ identification with and internalization of these cultures are directly related to whether the cultural function of the precinct can be truly realized and whether cultural communication can be effectively implemented. Yet the relationship between these Tourscape dimensions and tourists’ cultural identity has not been fully examined. This study responds to this gap, finding that in a completely newly built themed cultural precinct like Datang Everbright City, multiple dimensions of Tourscape are significantly associated with tourists’ cultural identity, indicating that even in the absence of authentic historical remains, Tourscape can still serve as an important context for tourists to identify with Tang culture.

Second, this study reveals the generative mechanism of cultural identity in themed cultural precincts, offering a new theoretical explanation for understanding “how modern landscapes carry historical meaning”. Drawing on constructive authenticity theory, the study finds that the socio-symbolic dimension is the strongest predictor of cultural identity. This indicates that in highly symbolic themed cultural precincts, tourists’ cultural identity does not necessarily depend on direct contact with historical remains but can be constructed and reinforced through symbolic elements in the Tourscape. Similarly, Chen et al. [60] taking the Angkor World Heritage Site in Cambodia as an example, found that symbolic consumption has a far greater influence on tourists’ place identity than functional and experiential consumption, indicating that symbol-level experiences also affect tourists’ deep psychological connections in authentic heritage sites. At the same time, this result also contrasts with the pathway of “objective authenticity → existential authenticity → loyalty” identified by Yi et al. in heritage tourism research [61]. In this study, the socio-symbolic dimension of Tourscape influences loyalty through the mediation of cultural identity. Both follow the basic logic of “environmental perception → psychological state → loyalty”, but the nature of the mediating mechanism differs. In traditional heritage tourism contexts, tourists’ psychological responses are more established upon the experience of the authenticity of historical remains; whereas in themed cultural precincts lacking authentic historical remains, cultural identity is formed more through the perception, experience, and consumption of symbolic elements in the Tourscape. This suggests that cultural meaning need not be exclusively attached to authentic heritage; it can also be reconstructed and gain tourist identification through the symbolic presentation of contemporary tourism scenes.

Third, drawing on the Tourscape framework, this study disaggregates the Stimulus in the S-O-R framework into multiple dimensions. Using cultural identity as the mediating link, it reveals how different types of environmental stimuli influence behavioral responses through differentiated psychological pathways. These findings help refine the application of the S-O-R framework in themed cultural spaces. The pathway pattern of this study forms a meaningful contrast with existing S-O-R research. Zhang and Xu [18], in their liminal experience study, found that the physical and social dimensions positively influence emotional arousal and liminal experience, with emotional arousal mediating the relationship between some Tourscape dimensions and liminal experience; whereas in this study, the social dimension exhibits both direct and indirect effects, with more diverse pathways. This indicates that even within the same cultural space, different dimensions of Tourscape influence tourists’ behavioral responses through distinct psychological pathways. In contrast, in traditional heritage tourism studies, environmental stimuli tend to influence behavioral intentions entirely through psychological states [62–66].

Meanwhile, our fieldwork also reveals that the natural dimension of Tourscape does not constitute a core source of tourists’ environmental perception in Datang Everbright City, a highly artificial themed cultural precinct. This finding contrasts with heritage or rural tourism settings, where nature often plays an important role [18,19], revealing the applicability limitations of the natural dimension in entirely artificial urban spatial contexts. In other words, the four-dimensional composition of the Tourscape framework is not fixed but is context-dependent, varying with the spatial attributes and landscape characteristics of the research object. This also suggests that future research should, when applying this framework, carefully select the dimensional composition according to the specific research context rather than mechanically applying all dimensions.

### Practical implications

These findings carry several practical implications for the scene construction and cultural identity cultivation of themed cultural precincts, challenging the logic of relying solely on customer satisfaction. Based on the findings, the following two suggestions are proposed. First, focus on the in-depth development of the socio-symbolic dimension to strengthen the symbolic driver of cultural identity. Block managers should shift their emphasis from large-scale physical landscape construction to the narrative integration and innovative transformation of cultural symbols. Specifically, it is necessary to systematically develop a highly recognizable creative product system, promote the extension of cultural IPs into deeply participatory experience projects, and enhance the depth of cultural narratives, facilitating tourists’ internalization of cultural values through consumption and interaction. As Ding et al. [13] pointed out, the power of themed spaces lies in transforming abstract cultural values into experiential, narrative, present-tense experiences for tourists. Managers of such blocks can more systematically integrate this concept into iterative design, transforming static cultural symbols into dynamic narrative consumption spaces.

Second, take cultural identity as the core mediating mechanism and systematically design the “scene-identity-behavior” transformation path, while fully utilizing the direct effect of the social dimension. The ultimate goal of scene creation is to foster deep meaning identification. Therefore, blocks should go beyond the pursuit of instantaneous traffic and embed participatory cultural narratives into the circulation routes and activities, transforming tourists from passive viewers into active meaning constructors, thereby deepening their experience from visual check-ins to emotional resonance and consolidating the behavioral basis for revisiting, recommending, and sharing. At the same time, the direct effect of the social dimension on loyalty suggests that high-quality performance interactions and immersive street performances can directly enhance tourist loyalty. Cai et al. [67] found in the context of nighttime tourism experiences that the experiencescape can effectively enhance tourist loyalty through place attachment. Datang Everbright City can further strengthen the symbolic recognizability of its signage system and cultural themed businesses on the basis of its existing light shows, combined with the enhanced effect of socio-symbolic elements during nighttime, so that “strolling through Everbright City” itself becomes an expressive consumption behavior of Tang cultural identity.

### Limitations and future directions

Several limitations of this study should be acknowledged.

First, the data of this study were collected through questionnaire surveys at a single point in time, which may present two limitations. On the one hand, the cross-sectional data limit causal inference; on the other hand, although we implemented some procedural remedies during the questionnaire design stage, the influence of common method bias cannot be completely ruled out. Future research could employ multi-source data for validation and further examine through longitudinal study designs whether tourists’ cultural identity changes with ongoing exposure to Tourscape and whether such changes further translate into stable loyalty behaviors.

Second, the generalizability of the findings is limited. On the one hand, as a single-case study, whether the conclusions from Datang Everbright City can be extended to other types of themed cultural attractions such as film studios or theme parks requires further empirical testing. On the other hand, the sample of this study was predominantly Chinese tourists and did not include tourists from other cultural backgrounds. As an important component of traditional Chinese culture, Tang culture holds special cultural affinity for Chinese tourists, which may strengthen the association between Tourscape dimensions and cultural identity. This effect may differ for tourists from other cultural backgrounds. Therefore, when applying the research model to themed cultural precincts in other countries and regions, it is necessary to fully consider differences in local tourists’ emotional connection and cultural distance with respect to specific cultural themes. Future research could validate our model across tourists from different cultural backgrounds to examine how cultural distance moderates the effects of each Tourscape dimension on cultural identity.

Third, the natural dimension of Tourscape was included in the pre-survey but excluded from the formal survey. This decision was primarily based on the spatial characteristics of the case site. Datang Everbright City is located in the urban core of Xi’an and is a highly artificial pedestrian precinct. Its core appeal derives from Tang-style architecture, thematic sculptures, light shows, and performing arts, while natural landscape elements serve only as background embellishments and do not constitute a core source of tourists’ environmental perception. We acknowledge that the sample size of the pre-survey was limited, but this does not affect the validity of the findings for the three retained dimensions. Future research will continue to examine this issue and would select themed cultural precincts with more prominent biophilic design as comparative cases to test the cross-contextual effects of the natural dimension.

In addition, data collection for this study was concentrated during the peak tourist season. Therefore, our findings primarily reflect tourists’ perceptual patterns under high-intensity experiential conditions during this period, and caution should be exercised when generalizing them to off-season contexts. During the off-season, visitor numbers are relatively lower, street performances are less frequent, and the overall atmosphere is less vibrant. Consequently, the direct effect of the social dimension on loyalty may weaken considerably, while the relationship between the socio-symbolic dimension and cultural identity may remain relatively more stable. Future research could incorporate off-season samples for cross-contextual comparisons to examine the moderating role of seasonality across different model paths, and further investigate the differences in tourists’ perception and identity formation processes under different experiential intensities.

## Conclusion

Taking Xi’an Datang Everbright City Pedestrian Street as a case and based on the Stimulus-Organism-Response theoretical framework, this study explored the mechanism through which different dimensions of Tourscape influence tourists’ destination loyalty via cultural identity. Based on analysis of 289 valid questionnaires using Partial Least Squares Structural Equation Modeling, the following main findings were obtained.

First, all three dimensions of Tourscape are significantly and positively associated with cultural identity. Among them, the socio-symbolic dimension has the strongest effect, followed by the physical and then social dimensions. This suggests that in a completely newly built immersive Tang cultural theme precinct such as Datang Everbright City, symbolic elements such as cultural symbols, creative products, and themed businesses are the primary source associated with tourists’ cultural identity, while visual spectacles and social interactions also play positive roles but with relatively weaker effects.

Second, cultural identity plays a mediating role between each dimension of Tourscape and destination loyalty, but the type of mediation varies. All three specific indirect paths are significant: the indirect effect of the socio-symbolic dimension on destination loyalty through cultural identity is 0.214, that of the physical dimension is 0.151, and that of the social dimension is 0.122. The direct effects of the physical and socio-symbolic dimensions are not significant, indicating that cultural identity fully mediates the relationship; the direct effect of the social dimension is significant, indicating a partial mediating role. This finding confirms that cultural identity is the key psychological channel connecting Tourscape and tourists’ behavioral intentions, while also revealing the differences in the mechanisms of the three dimensions.

Third, the direct effects of the three Tourscape dimensions on destination loyalty differ significantly. The direct effect of the social dimension is significant, while the direct effects of the physical and socio-symbolic dimensions are not significant. This indicates that in themed cultural precincts, social interaction and performance experiences are directly associated with tourists’ revisit and recommendation intentions without fully relying on deep cultural identity; in contrast, the physical environment and cultural symbols are associated with loyalty behavior through the internalization process of cultural identity.
